# The effect of school support on vocational college teachers’ digital literacy: the moderating role of professional title and the mediating role of professional identity and psychological empowerment

**DOI:** 10.3389/fpsyg.2026.1866813

**Published:** 2026-06-17

**Authors:** Yiwen Zhang, Yigang Lin, Fangyan Chen, Tinglai Chen, Junsheng Cao, Xiaoyu Cheng

**Affiliations:** 1 College of Education, Zhejiang Normal University, Jinhua, China; 2 School of Education, Jinhua University of Vocational Technology, Jinhua, China

**Keywords:** digital literacy, professional identity, psychological empowerment, serial mediation, vocational college teachers

## Abstract

In the context of vocational education’s digital transformation, enhancing teachers’ digital literacy is critically important. However, existing research has focused primarily on external interventions such as infrastructure and training, often overlooking digital literacy as a “professional ability” requiring long-term cultivation that fundamentally depends on teachers’ internal psychological motivation. Specifically, how school support translates into improved digital literacy through teachers’ psychological states remains underexplored, particularly among vocational college teachers. To address this gap, this study proposed a serial mediation model linking school support, psychological empowerment, professional identity, and digital literacy. Data from 493 vocational college teachers revealed that school support significantly and positively predicted digital literacy. Both psychological empowerment and professional identity partially mediated this relationship, with a significant serial mediation effect also present. While professional title did not moderate the overall mediation mechanism, distinct differences emerged in the mediation pathways across title groups. These findings offer empirical support for developing stratified digital literacy training systems for vocational college teachers, with important implications for fostering high-quality, intrinsic development in vocational education during digital transformation.

## 1 Introduction

The rapid development of digital transformation and artificial intelligence has made enhancing teachers’ digital literacy a global consensus and a key education reform initiative. Organizations such as the European Union (European Commission, Joint Research Centre, 2017) and United Nations Educational, Scientific and Cultural Organization (2018) have issued digital competence frameworks outlining essential competencies for educators. In response, the Ministry of Education of the People’s Republic of China (2022) released the “Teacher Digital Literacy” industry standard, followed by the “Circular on Organizing and Implementing the Digital Empowerment Action for Teacher Development” (Ministry of Education of the People’s Republic of China, 2025). Within this strategic framework, the digital transformation of vocational education has acquired particular significance and urgency. According to the 2024 National Education Development Statistical Bulletin (Ministry of Education of the People’s Republic of China, 2024), vocational colleges account for over 51% of all higher education institutions. More importantly, as the education sector most closely linked to industrial and economic development (Ren and Zhou, 2022), vocational education bears the core mission of cultivating high-quality technical talent (Chen et al., 2022), master craftsmen, and national-level artisans (Wang et al., 2022). Thus, the digital literacy of vocational teaching staff not only influences instructional innovation but also directly affects talent development quality and the depth of industry-education integration.

However, existing empirical research has predominantly focused on digital literacy among primary, secondary, and general higher education teachers, including specific disciplines (Ayyildiz et al., 2021; Lei and Jiang, 2025; Lyu and Luo, 2024; Wang and Ye, 2025; Zhang, 2023), while vocational education remains underrepresented. Furthermore, most studies examine single-dimensional factors, either external environmental variables such as school support (Chen et al., 2025) or individual cognitive factors like self-efficacy (Cosby et al., 2023; Feng et al., 2024), without integrating environmental and personal, objective and subjective factors into a unified theoretical framework to systematically examine their interactive mechanisms.

To address these gaps and clarify the pathways to enhancing digital literacy among vocational college teachers, this study drew on Bandura’s social learning theory to construct an integrated “environment-psychology-behavior” model (Bandura, 1977). The model positions school support as the environmental variable, with psychological empowerment and professional identity serving as individual psychological mediators, offering a systematic theoretical framework for understanding and intervening in vocational teachers’ digital literacy development.

Specifically, this study makes three contributions. First, it integrates psychological empowerment and professional identity as serial mediators linking school support to digital literacy among vocational college teachers—a mechanism previously unexamined in this context. Second, it moves beyond average effects by investigating how professional title moderates these pathways, responding to calls for stage-sensitive teacher development models. Third, by focusing on vocational college teachers, an under-researched but strategically important group, this study extends digital literacy research from general education to the vocational sector, providing empirical evidence for differentiated policy interventions.

## 2 Theoretical background and hypotheses

### 2.1 School support and digital literacy

In the context of educational digital transformation, teacher digital literacy is widely regarded as a key factor influencing technology-enabled education. In recent years, the connotation of digital literacy has continuously expanded. It no longer merely focuses on the proficiency in operating digital tools but further encompasses meta cognitive abilities as well as social and emotional capabilities, thereby enabling educators to achieve continuous innovation in the rapidly evolving digital ecosystem (Kamsker et al., 2020; Liu et al., 2025; Ng, 2012; Nguyen and Habók, 2024; Wang and Lu, 2021; Williamson et al., 2019).

Perceived organizational support (POS) refers to the extent to which employees believe that their organization values their work contributions and is concerned about their personal welfare. In other words, the degree to which they feel supported across multiple dimensions (Eisenberger et al., 1986). In the school context, organizational support denotes teachers’ subjective perceptions of the support provided by their institution, typically encompassing infrastructure, technological tools, resource platforms, and institutional safeguards. According to organizational support theory, providing instrumental support (i.e., equipment, information, and training) enables organizations to meet employees’ work conditions and developmental opportunities, thereby directly enhancing work performance (McMillan, 1997). Empirical evidence supports this theoretical perspective: digital tools, practice platforms, and training support provided by schools serve as key work resources that help teachers master digital technologies, thereby enhancing their digital literacy and professional capabilities (Chiu et al., 2024; Simbula et al., 2011). Therefore, this study proposes the following hypothesis:

*H1*: School support positively predicts the digital literacy of vocational college teachers.

### 2.2 School support, psychological empowerment, and digital literacy

Psychological empowerment refers to the mental state in which an individual perceives having control over their work (Spreitzer, 2008). It is generally regarded as an internal motivational mechanism that profoundly influences individual behavior (Kong et al., 2016). According to Spreitzer (1995), psychological empowerment comprises four dimensions: meaning, competence, self-determination, and impact. Drawing on Self-Determination Theory (Deci and Ryan, 2000), individuals develop high-quality intrinsic motivation when their three fundamental psychological needs (i.e., autonomy, competence, and relatedness) are satisfied, thereby actively engaging in learning and practice. In the context of vocational education, psychological empowerment reflects teachers’ positive experiences of these core needs: autonomy represents control over teaching decisions; competence manifests as confidence in applying digital technologies; and meaning relates to professional value recognition and group belonging. When vocational teachers experience heightened empowerment, their intrinsic motivation is effectively stimulated, making them more inclined to proactively explore, engage in continuous learning, and creatively integrate digital tools into teaching, thereby systematically enhancing their digital literacy. Empirical evidence supports this theoretical perspective. Mantegh and Jabbari (2021) found that psychological empowerment not only significantly and positively predicts teachers’ digital literacy, but its three dimensions—competence, autonomy, and impact—also exhibit significant positive relationships with digital literacy.

Furthermore, perceived organizational support (POS) has been shown to positively predict employees’ psychological empowerment (Afzali et al., 2014; Caesens et al., 2020; Iqbal and Hashmi, 2015; Maan et al., 2020). As Özaralli (2003) emphasized, employees who feel supported by their organization tend to believe they can complete their tasks—a sense of competence that constitutes one of the core dimensions of psychological empowerment (Thomas and Velthouse, 1990). In a survey of Chinese language teachers, perceived organizational support was found to predict higher psychological empowerment (Yu et al., 2024). Thus, when teachers perceive development opportunities and emotional care provided by their schools, they are more likely to feel affirmed and recognized, thereby experiencing higher levels of psychological empowerment. Based on the theoretical foundations and empirical evidence reviewed above, this study proposes the following hypotheses:

*H2*: School support has a significant positive effect on psychological empowerment.

*H3*: Psychological empowerment has a significant positive effect on teachers’ digital literacy.

*H4*: Psychological empowerment mediates the relationship between school support and teachers’ digital literacy.

### 2.3 School support, professional identity, and digital literacy

Teacher professional identity refers to the positive cognition, experiences, and behavioral tendencies that teachers hold toward their profession and internalized professional role, which is essentially a positive attitude related to the teaching profession (Wei, 2008). Social identity theory (Tajfel and Turner, 1979) emphasizes that the degree to which individuals identify with their professional identity is influenced by the social environment. When individuals perceive that they are respected, supported, and valued, they identify more strongly with that identity and exhibit behaviors consistent with it. Extensive research indicates that when organizations provide support and confer status upon a professional group, members’ identification with that profession significantly increases (Ashforth and Mael, 1989; Flores and Day, 2006; Li et al., 2026; Song et al., 2024; Zhang et al., 2025). School support precisely represents an affirmative signal from the external environment toward the professional group of “vocational college teachers”. When schools provide adequate support, teachers perceive that their professional roles are valued and recognized by the organization, thereby strengthening their self-concept as “vocational educators”. This enhanced professional identity further motivates teachers to actively participate in professional development activities to maintain the consistency and integrity of their identities. Previous studies have shown that primary and secondary school teachers’ professional identity significantly and positively predicts their digital literacy (Wang and Ye, 2025). Based on this reasoning, the following hypotheses are proposed:

*H5*: School support has a significant positive effect on professional identity.

*H6*: Professional identity has a significant positive effect on teachers’ digital literacy.

*H7*: Professional identity mediates the relationship between school support and teachers’ digital literacy.

### 2.4 School support, psychological empowerment, professional identity, and digital literacy

Social cognitive theory (Bandura, 2001) posits that individuals shape their self-concept through cognitive assessment, which in turn guides behavioral choices and engagement. When teachers experience a high level of psychological empowerment, their perception of the “teacher role” undergoes positive reconfiguration: from passive executor to competent and influential professional. This proactive cognitive awareness not only strengthens their belief in their own capabilities and the controllability of the environment (Conger and Kanungo, 1988) but also enhances their tendency to internalize the identity of “vocational educator.” Positive beliefs such as self-efficacy and perceived environmental support significantly enhance individuals’ behavioral motivation and work engagement, thereby promoting professional identity development (Bakker et al., 2005). Thus, when teachers are persistently energized by intrinsic motivation and positive psychological resources obtained from their work, they are more likely to perceive alignment between their expectations and the working environment, enhancing their sense of self-worth and professional identity. Empirical research consistently demonstrates that psychological empowerment positively predicts professional identity: educators with stronger empowerment experiences exhibit more pronounced professional identity (Ding and Xie, 2021; Fox et al., 2015). Based on the above reasoning, this study proposes the following hypotheses:

*H8*: Psychological empowerment has a significant positive effect on professional identity.

*H9*: Psychological empowerment and professional identity serve as serial mediators in the relationship between school support and digital literacy.

### 2.5 Moderating effects of teacher professional titles

Although school support may enhance vocational teachers’ digital literacy through psychological empowerment and professional identity, this mechanism may vary systematically depending on teachers’ career stages. Huberman (1993) proposed that a teacher’s career can be divided into several stages: entry, stabilization, experimentation and diversification, serenity, and disengagement. Teachers at different stages exhibit significant differences in their role focus, professional demands, and developmental tasks. Junior-title teachers concentrate on role adaptation and skill accumulation, intermediate teachers focus on identity establishment and professional influence, while senior-title teachers place greater emphasis on independent innovation and experience transmission.

Meanwhile, conservation of resources theory (Hobfoll, 1989) contends that individuals seek to accumulate, maintain, and defend resources they value, such as time, expertise, and social connections. Senior-level teachers typically possess richer “resource reserves,” including professional reputation, technical expertise, and institutional influence, enabling them to more efficiently convert external support into intrinsic motivation. In contrast, junior-level teachers, constrained by limited resources, rely more on meaning construction mechanisms, such as professional identity, to maintain their engagement motivation. A study on special education teachers also demonstrated that teaching experience significantly affects professional identity, revealing a nonlinear distribution across experience groups: teachers with over 15 years of experience exhibited the strongest identity, followed by those with less than 5 years, while those with 5 to 15 years showed relatively lower professional identity levels. Differences among the three groups reached statistical significance (Hong, 2025).

Therefore, professional title serves as a key indicator of professional credentials, organizational status, and psychological needs, profoundly influencing individuals’ perceptions, internalization, and behavioral transformation of support resources. Accordingly, this study proposes the following hypotheses:

*H10*: Professional title groups may exert a moderating effect on the overall mediating mechanism involving school support, psychological empowerment, professional identity, and digital literacy.

**Figure 1:**
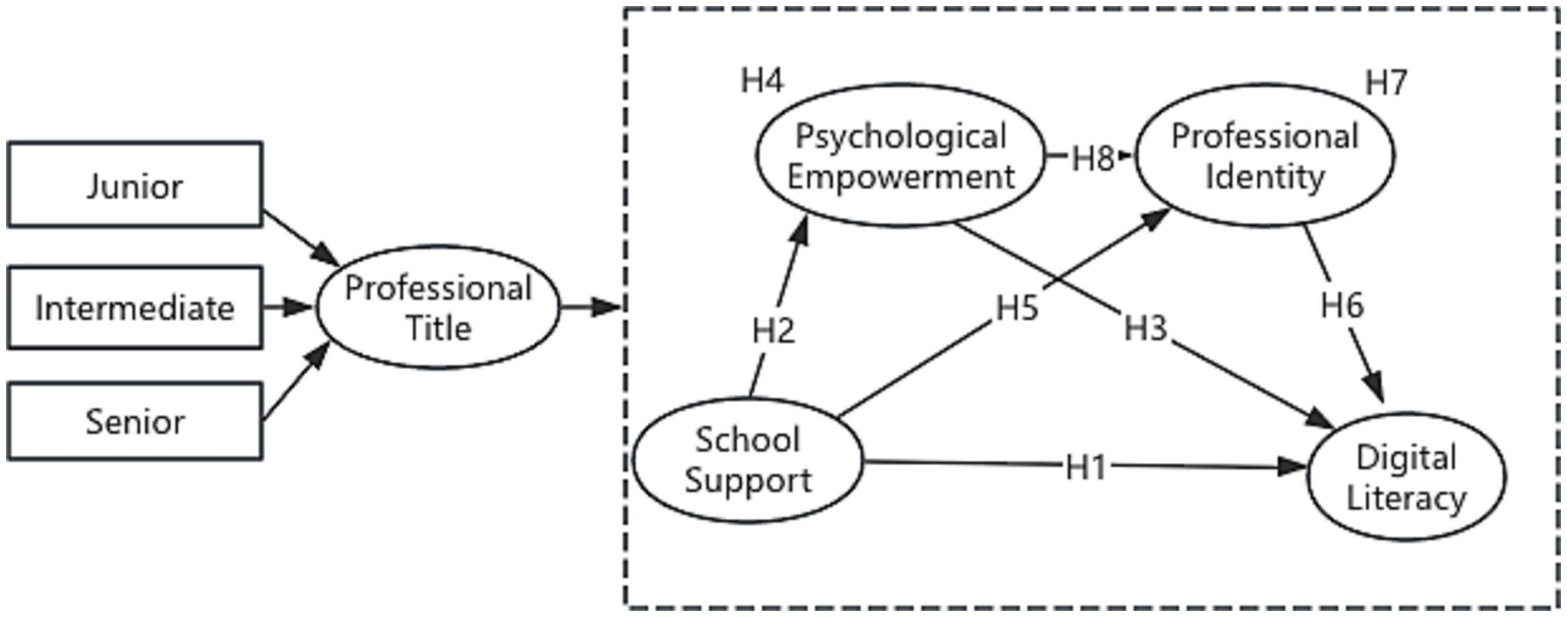
Hypothesized model pathways (inside the dotted line is the mediating assumption, outside the dotted line is the moderating assumption).

The hypothesized model is depicted in Figure 1.

## 3 Materials and methods

### 3.1 Participants

Participants were recruited using a multi-stage stratified sampling strategy. First, based on China’s three major economic regions (eastern, central, and western), two provincial-level administrative units were randomly selected from each region, resulting in six target provinces. Second, within each selected province, three public vocational colleges with which the research team had prior collaborative relationships were randomly drawn, yielding a total of 18 target institutions. Third, the academic affairs offices of these institutions were contacted, and a total of 520 questionnaires were distributed through the Wenjuanxing platform to invite full-time vocational college teachers to participate voluntarily. Before the survey, participants were provided with an explanation of the research purpose and assured of data confidentiality. After data collection, responses with abnormal completion times (i.e., less than 120 s), patterned responses, and invalid questionnaires were excluded, yielding 493 valid responses, representing an effective response rate of 94.8%. Table 1 presents the detailed demographic information of the participants.

**Table 1:** Demographic characteristics of participants (*N* = 493).

Variables	Category	*N*	*%*
Gender	Male	176	35.7
	Female	317	64.3
School location	Eastern region	195	39.6
	Central region	180	36.5
	Western region	118	23.9
Professional title	Junior	181	36.7
	Intermediate	186	37.7
	Senior	126	25.6
Subject taught	Humanities and social sciences	315	63.9
	Science and engineering	178	36.1
Educational background	Bachelor’s degree or below	198	40.2
	Master’s degree	258	52.3
	Doctor’s degree	37	7.5

### 3.2 Measurements

The measurement tools consisted of two sections. The first section collected demographic information, including school location, gender, age, professional title, subject taught, and educational background. The second section comprised the School Support Questionnaire, Psychological Empowerment Scale, Professional Identity Scale, and Digital Literacy Questionnaire.

#### 3.2.1 School support

The adaptation of the School Support Questionnaire for Vocational College Teachers originates from the Enabling Conditions Scale developed by Venkatesh et al. (2003). Its rigor and professionalism have been recognized and applied by domestic scholars (Wang and Chu, 2023). In this study, the scale was appropriately modified to align with the contextual characteristics of vocational college teachers. It consists of 8 items, including statements such as ‘The school where I work has policies and systems to enhance teachers’ digital capabilities to support the development of our digital skills’. Responses were evaluated using a five-point Likert scale, ranging from 1 (*strongly disagree*) to 5 (*strongly agree*). The higher the score, the greater the level of support provided by the school for vocational college teachers.

In this study, the Cronbach’s *α* coefficient for the questionnaire was 0.943. The average variance extracted (*AVE*) was 0.683, and the composite reliability (*CR*) was 0.945, indicating good convergent validity. Additionally, the confirmatory factor analysis showed acceptable model fit: *χ*^2^*/df* = 2.1, *CFI* = 0.99, *TL*I = 0.99, *IFI* = 0.99, *RMSEA* = 0.05. Given that the scale is unidimensional and consists of only eight items, these results indicate that this scale possesses good reliability and validity and is suitable for use with the participants in this study.

#### 3.2.2 Psychological empowerment

The Psychological Empowerment Scale, developed by Spreitzer (1995), was employed in this study. This scale has been widely used in research measuring psychological empowerment, with its reliability and validity well established (Aziz et al., 2024; Ochoa Pacheco and Coello-Montecel, 2023; Yogalakshmi and Suganthi, 2020). The scale comprises four dimensions: work meaningfulness, competence, self-determination, and influence. It consists of 12 items, including statements such as “The work I do is very meaningful to me.” All items were measured on a five-point Likert scale where 1 = “*strongly disagree,*” and 5 = “*strongly agree*,” with higher scores indicating stronger psychological empowerment among vocational college teachers.

The overall Cronbach’s *α* coefficient for the scale was 0.905. The *AVE* was 0.512, and the *CR* was 0.802, indicating acceptable convergent validity. The Cronbach’s α coefficients for the four subscales were 0.898, 0.876, 0.936, and 0.897, respectively, demonstrating good internal consistency. Confirmatory factor analysis indicated an acceptable model fit: *χ*^2^*/df* = 3.658, fit indices *CFI* = 0.973, *TLI =* 0.963, *RMSEA* = 0.074, collectively confirming the sound structural validity of the scale.

#### 3.2.3 Professional identity

The Vocational Teacher Professional Identity Scale used in this study was adapted from the Teacher Professional Identity Scale originally developed by Wei et al. (2013). This scale has demonstrated high reliability and validity in multiple studies (Ding and Xie, 2021; Lu et al., 2022; Xiang et al., 2024). In this study, appropriate modifications were made to the scale, which comprises four dimensions: role values, behavioral tendencies, professional values, and sense of belonging. It consists of 18 items, including statements such as “I believe the work of vocational college teachers plays an important role in the development of human society.” All items are measured on a five-point Likert scale (1 = strongly *disagree*, 5 = *strongly agree*). Higher scores indicate a greater level of professional identity among vocational college teachers.

The overall Cronbach’s *α* coefficient for the scale was 0.966. The *AVE* was 0.791, and the *CR* was 0.985, indicating good convergent validity. Additionally, the Cronbach’s α coefficients for the four subscales were 0.944, 0.936, 0.922, and 0.820, respectively. Confirmatory factor analysis indicate an excellent model fit: *χ*^2^*/df* = 2.05, *CFI* = 0.99, *TLI* = 0.98, *IFI* = 0.99, *RMSEA* = 0.05. Overall, the scale demonstrated good reliability and validity.

#### 3.2.4 Digital literacy

The Digital Literacy Questionnaire for Vocational College Teachers adopted the Digital Literacy Scale developed by Ng (2012). Huang (2023) confirmed that this scale has high reliability and validity. The questionnaire consists of 10 items, including statements such as “I know how to solve my own technical problems.” A five-point Likert scale was used to quantitatively evaluate the digital literacy level of vocational college teachers. The self-assessment scores ranged from 1 (strongly *disagree*) to 5 (*strongly agree*), with higher scores indicating a higher level of digital literacy.

In this study, the Cronbach’s α coefficient for the scale was 0.947. The *AVE* was 0.693, and the *CR* was 0.957, indicating good convergent validity. Additionally, the results of confirmatory factor analysis showed a good model fit: *χ*^2^*/df* = 1.77, *CFI* = 0.99, *TLI* = 0.99, *IFI* = 0.99, and *RMSEA* = 0.04. Given that the scale is unidimensional and consists of only 10 items, these fit indices suggest that the scale demonstrates good reliability and validity within the context of this study.

### 3.3 Procedure

This study was approved by the ethics review committee of the authors’ institution. Data were collected through an online questionnaire platform.^1^ After providing informed consent, which assured anonymity, confidentiality, and the right to withdraw at any time, all participants completed a questionnaire that included demographic variables, school support, digital literacy, psychological empowerment, and professional identity. The average completion time was approximately 15 min. Upon submission, participants were debriefed on the study’s purpose and thanked for their participation.

### 3.4 Statistical analysis

Firstly, this study employed SPSS 26.0 to conduct descriptive analyses of valid responses, test the reliability and validity of the scales, and examine correlations among core variables using Pearson’s correlation coefficient. Secondly, AMOS 26.0 was used to perform confirmatory factor analysis, construct the serial mediation model, and assess model fit. Based on the satisfactory model fit, an in-depth analysis of the relationships among core variables was conducted. Thirdly, the bias-corrected nonparametric percentile bootstrap method was employed to calculate path coefficients and their confidence intervals, thereby testing the serial mediation hypotheses. Finally, multi-group analysis was conducted to compare each mediation path across different professional title groups.

## 4 Results

### 4.1 Preliminary analyses

#### 4.1.1 Common method bias

Control of common method bias typically follows two major strategies: procedural control and statistical control (Zhou and Long, 2004). To mitigate its impact at the source, this study implemented procedural control measures during the design and administration stages (Podsakoff et al., 2003). These included ensuring respondent anonymity, incorporating reverse-scored items, and informing participants that all data would be used solely for academic research purposes, thereby enhancing response authenticity. In the data analysis stage, this study further employed Harman’s single-factor test method for statistical control (Podsakoff et al., 2003). The results revealed seven common factors with eigenvalues greater than one, with the first factor accounting for 38.47% of the total variance, below the threshold of 40%. Based on the results of both procedural and statistical controls, common method bias does not pose a serious concern in this study.

#### 4.1.2 Basic analysis

Table 2 presents the means, standard deviations, and correlations for the core variables. The results show significant positive correlations among these variables, providing preliminary support for subsequent mediation analysis.

**Table 2:** Descriptive statistics and correlations among the major variables.

Variables	PE	PI	DL	SS
PE	1			
PI	0.425^**^	1		
DL	0.346^**^	0.235^**^	1	
SS	0.458^**^	0.387^**^	0.436^**^	1
M	3.723	4.146	3.674	3.73
SD	0.934	0.75	0.75	0.716

PE means psychological empowerment; PI means professional identity; DL means digital literacy; SS means school support. M and SD means Mean and standard deviation, respectively. **p < 0.01.

#### 4.1.3 Assessment of model fit

The higher the model fit, the stronger the model’s validity, and the greater the substantive meaning of the parameter values. This study employed AMOS 26.0 to test the serial mediation model. The model fit indices were as follows: *χ*^2^*/df* = 3.929, *GFI* = 0.831, *AGFI* = 0.795, *PGFI* = 0.686, *PNFI* = 0.801, *SRMR* = 0.055, *RMSEA* = 0.077. The results indicate that all fit indices meet the recommended thresholds of Hu and Bentler (1999), indicating an acceptable model fit and supporting further interpretation of the path coefficients.

### 4.2 Path analysis

**Figure 2:**
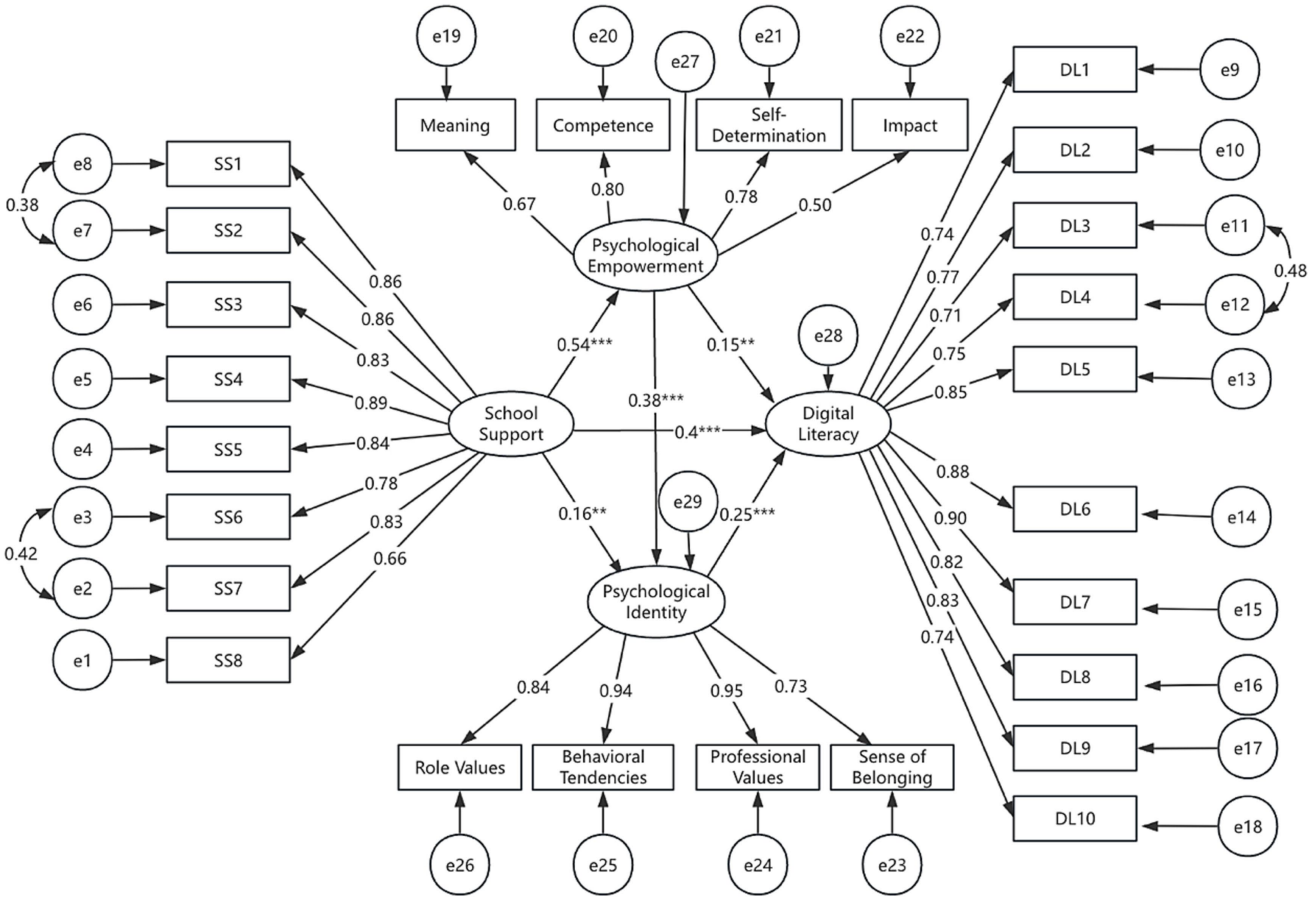
Serial mediation model with standardized path coefficients (^**^p < 0.01, ^***^p < 0.00.1).

Path analysis was conducted to examine the hypothesized relationships among school support, psychological empowerment, professional identity, and digital literacy. As shown in Figure 2, all standardized path coefficients ranged from 0.15 to 0.54, well below the threshold of 1, satisfying the basic requirements for parameter estimation in structural equation modeling (Kline, 2023). These results indicate that the model demonstrated good fit, with all paths being statistically significant. The hypothesis testing results are presented in Table 3. School support had a significant positive effect on the digital literacy of teachers (*β* = 0.404, *p* < 0.001), psychological empowerment (*β* = 0.544, *p* < 0.001), and professional identity (*β* = 0.165, *p* = 0.003), with the strongest effect observed on psychological empowerment. Psychological empowerment significantly predicted digital literacy (*β* = 0.148, *p* = 0.007) and professional identity (*β* = 0.381, *p* < 0.001), with a particularly pronounced effect on the latter. Professional identity also exerted a significant positive effect on digital literacy (*β* = 0.249, *p* < 0.001). Accordingly, hypotheses H1, H2, H3, H5, H6, and H8 were all supported.

**Table 3:** Path coefficients for the structural equation model.

Hypotheses	Paths	Estimate	S.E.	C.R.	*p*
H1	SS → DL	0.404	0.051	7.441	< 0.001
H2	SS → PE	0.544	0.051	9.035	< 0.001
H3	PE → DL	0.148	0.062	2.691	< 0.01
H5	SS → PI	0.165	0.045	2.999	< 0.01
H6	PI→DL	0.249	0.054	5.401	< 0.001
H8	PE → PI	0.381	0.06	6.079	< 0.001

SS means school support; PE means psychological empowerment; PI means professional identity; DL means digital literacy. Estimate means Standardized Estimate. S.E. means Standard Error; C.R. means Critical Ratio.

### 4.3 Testing for serial mediation effects

To examine the mediating roles of psychological empowerment and professional identity, and to test the serial mediation hypotheses (H4, H7, and H9) concerning the relationship between school support and digital literacy among vocational college teachers. This study applied the bias-corrected nonparametric percentile bootstrap method, generating 5,000 resamples to estimate mediation effects and their 95% confidence intervals (Preacher and Hayes, 2008). As presented in Table 4, all three mediation hypotheses were supported. Firstly, the total effect of school support on digital literacy was significant, with an effect value of 0.578, and a 95% confidence interval excluding zero (Hayes, 2018). The direct effect value was also significant [*β* = 0.404, *95% CI* = (0.321, 0.479)], indicating that school support directly predicts digital literacy even after accounting for mediators. Secondly, regarding the specific mediating pathways, psychological empowerment independently mediated the relationship between school support and digital literacy, with a significant indirect effect of 0.081 [*95% CI =* (0.028, 0.136)], accounting for 46.55% of the total effect. Professional identity also served as a significant independent mediator, with an indirect effect of 0.041 [*95% CI* = (0.016, 0.077)], explaining 23.56% of the total effect. Finally, the serial mediation effect from psychological empowerment to professional identity was also significant, with an indirect effect of 0.052 [*95% CI* = (0.034, 0.081)], accounting for 29.89% of the total effect. These findings demonstrate that school support enhances vocational college teachers’ digital literacy not only directly but also indirectly through the serial mediation of psychological empowerment and professional identity.

**Table 4:** Bootstrap test of standardized mediation effects.

Path	Estimate	*95% CI*		*p*
		Lower	Upper	
Total effect	0.578	0.514	0.64	< 0.001
Direct effect	0.404	0.321	0.479	< 0.001
Total mediating effect	0.174	0.123	0.23	< 0.001
SS → PE → DL	0.081	0.028	0.136	< 0.01
The proportion of the mediating effect of PE	46.55%			
SS → PI→DL	0.041	0.016	0.077	< 0.01
The proportion of the mediating effect of PI	23.56%			
SS → PE → PI→DL	0.052	0.034	0.081	< 0.001
The proportion of the serial effect	29.89%			

SS means school support; PE means psychological empowerment; PI means professional identity; DL means digital literacy.

### 4.4 Multi-group comparison based on teacher professional titles

To test H10, this study employed multi-group structural equation modeling (MGSEM) to examine structural invariance across professional titles. As shown in Table 5, based on the unconstrained model (Model 1), serially imposing constraints on measurement weights (Model 2) and structural weights (Model 3) resulted in non-significant changes in model fit (Δ*χ*^2^ *M2-M1* = 54.504, *p* > 0.05; Δ*χ*^2^ *M3-M1* = 74.282, *p* > 0.05). These results indicate that the proposed theoretical model demonstrates measurement and structural invariance across junior, intermediate, and senior professional title groups. Therefore, H10 was not supported.

**Table 5:** Fit indices for multi-group invariance testing across professional titles.

Model	*χ^2^*	*df*	*χ^2^/df*	*TLI*	*CFI*	*RMSEA*	*ΔCFI*	*ΔRMSEA*	*Δχ^2^*	*Δdf*	*p*
M1 Unconstrained	1970.279	870	2.265	0.889	0.901	0.051					
M2 Measurement weights	2024.783	914	2.215	0.893	0.9	0.05	−0.001	−0.001	54.504	44	0.133
M3 Structural weights	2044.561	926	2.208	0.894	0.899	0.05	−0.002	0	74.282	56	0.052

However, the overall universality of the model does not preclude the possibility of local heterogeneity in specific pathways or mediating mechanisms. Therefore, after verifying cross-group comparability, this study further estimated structural paths and mediating effects for each of the three professional title subsamples. The bootstrap method was employed to calculate standardized indirect effects and their 95% confidence intervals. As shown in Table 6, the path “School Support → Professional Identity” was significant only in the intermediate-level group, while the path “Psychological Empowerment → Digital Literacy” was significant only in the senior-level group. Regarding the mediating pathways: The mediating effect of psychological empowerment was significant only in the senior-level group [*95% CI* = (0.013, 0.27)], but not in the junior-level [*95% CI* = (−0.003, 0.21)] and intermediate-level groups [*95% CI* = (−0.088, 0.167)]. The mediating effect of professional identity was significant only in the intermediate-level group [*95% CI* = (0.002, 0.114)], but not in the junior-level [*95% CI* = (−0.017, 0.157)], and senior-level groups [*95% CI* = (−0.066, 0.074)]. The serial mediation effect was significant only in the junior-level group [*95% CI* = (0.038, 0.176)], but not in the intermediate-level [*95% CI* = (−0.002, 0.049)] and senior-level groups [*95% CI* = (−0.002, 0.102)].

**Table 6:** Standardized path coefficients and confidence intervals for mediation effects across professional title groups.

Path	Junior		Intermediate		Senior	
	*β*	*95% CI*	*β*	*95% CI*	*β*	*95% CI*
SS → PE	0.53	[0.391, 0.668]	0.557	[0.418, 0.702]	0.546	[0.354, 0.699]
SS → PI	0.146	[−0.057, 0.359]	0.33	[0.16, 0.517]	0.002	[−0.238, 0.358]
PE → PI	0.476	[0.244, 0.674]	0.232	[0.014, 0.409]	0.407	[0.096, 0.619]
PE → DL	0.175	[−0.007, 0.342]	0.058	[−0.155, 0.277]	0.278	[0.021, 0.504]
PI→DL	0.368	[0.208, 0.524]	0.149	[0.007, 0.297]	0.191	[−0.009, 0.38]
SS → PE → DL	0.093	[−0.003, 0.21]	0.032	[−0.088, 0.167]	0.151	[0.013, 0.27]
SS → PI→DL	0.054	[−0.017, 0.157]	0.049	[0.002,0.114]	0	[−0.066,0.074]
SS → PE → PI→DL	0.093	[0.038, 0.176]	0.019	[−0.002, 0.049]	0.042	[−0.002, 0.102]
Direct effect	0.372	[0.194, 0.522]	0.447	[0.267, 0.612]	0.338	[0.096, 0.538]
Total mediating effect	0.239	[0.126, 0.36]	0.101	[−0.016, 0.236]	0.194	[0.041, 0.35]
Total effect	0.611	[0.464, 0.729]	0.548	[0.397, 0.68]	0.532	[0.349, 0.691]

To further examine the professional title in the mediating mechanism, this study employed multi-group structural equation modeling (MGSEM) and the bootstrap difference test to conduct cross-group comparisons of the three mediating pathways. As shown in Table 7: on the psychological empowerment mediation pathway, the indirect effect of senior-level teachers [*β* = 0.151, *95% CI =* (0.013, 0.27)] was significantly higher than that for intermediate-level teachers [*β* = 0.032, *95% CI* = (−0.088, 0.167)], with a difference of 0.119, *95% CI* = [0.009, 0.229]. This indicates that senior-level teachers are more capable of converting school support into digital literacy through psychological empowerment. On the professional identity mediation pathway, although the indirect effect for intermediate-level teachers was significant [*β* = 0.049, *95% CI* = (0.002, 0.114)], the effects for junior teachers [*β* = 0.054, *95% CI* = (−0.017, 0.157)] and senior-level teachers [*β* = 0.000, *95% CI =* (−0.066, 0.070)] were not significant. However, the confidence intervals for all group differences included zero [the effect difference between intermediate and junior-level teachers was 0.005, *95% CI* = (−0.100, 0.090), and the effect difference between intermediate and senior-level teachers was 0.049, 95% *CI =* (−0.050, 0.148)], indicating that the group differences on this path did not reach statistical significance. On the serial mediation pathway, the indirect effect for junior-level teachers [*β* = 0.093, *95% CI =* (0.038, 0.176)] was significantly higher than that of intermediate-level teachers [*β* = 0.019, *95% CI =* (−0.002, 0.049)], with a difference of 0.074 [*95% CI =* (0.001, 0.148)]. This suggests that junior-level teachers rely more on the complete “empowerment-identity” serial mediation pathway, whereas this mechanism has weakened for intermediate-level teachers.

**Table 7:** Test of between-group differences in mediating effects across professional titles.

Path	Group	*(Δ)*	*95% CI*	Results
SS → PE → DL	Senior vs. Intermediate	0.119	[0.009, 0.229]	Supported
	Senior vs. Junior	0.058	[−0.109, 0.225]	Unsupported
	Junior vs. Intermediate	0.061	[−0.115, 0.237]	Unsupported
SS → PI→DL	Intermediate vs. Junior	−0.005	[−0.100, 0.090]	Unsupported
	Intermediate vs. Senior	0.049	[−0.050, 0.148]	Unsupported
	Junior vs. Senior	0.054	[−0.095, 0.203]	Unsupported
SS → PE → PI→DL	Junior vs. Intermediate	0.074	[0.001, 0.148]	Supported
	Junior vs. Senior	0.051	[−0.049, 0.151]	Unsupported
	Intermediate vs. Senior	−0.023	[−0.112, 0.066]	Unsupported

## 5 Discussion

This study finds that school support positively predicts teachers’ digital literacy. Psychological empowerment and professional identity not only independently mediate this relationship but also function as serial mediators. We also identified significant differences in these mediating paths across teachers with different professional titles. The results are discussed below.

### 5.1 The impact of school support on the digital literacy among vocational college teachers

The results indicate that school support has a significant positive predictive effect on the improvement of digital literacy among vocational college teachers, which aligns with previous research. Ochoa Pacheco and Coello-Montecel (2023), through surveys of primary and secondary school teachers, demonstrated that school support, such as hardware provision and training opportunities, significantly and positively predicts teachers’ digital literacy. In other words, organizational resources, institutional guarantees, and leadership support can effectively remove barriers to digital technology application, stimulate teachers’ intrinsic learning motivation and practical enthusiasm, and thereby promote the development of their digital literacy. This finding is consistent with the core tenets of social ecosystem theory (Bronfenbrenner, 1979). The development of individuals is influenced by multi-level environmental systems, particularly the microsystems with which they interact directly. For vocational college teachers, the school environment serves as a critical microsystem that fosters digital literacy development by creating a supportive and empowering ecological atmosphere.

Specifically, in terms of institutional support, vocational colleges should integrate digital literacy into job responsibilities, performance evaluations, and professional criteria, while establishing incentive mechanisms. At the resource level, schools should develop digital teaching environments that integrate industry and education, and introduce enterprise-level digital tools, thereby ensuring continuous access to high-quality digital resources and providing structural support for enhancing teachers’ digital literacy.

### 5.2 The mediating role of psychological empowerment in relationships

Although existing studies have separately demonstrated that perceived organizational support positively predicts psychological empowerment (Song et al., 2024; Yu et al., 2024) and that psychological empowerment significantly enhances teachers’ digital literacy (Mantegh and Jabbari, 2021), no research has yet integrated these three constructs into a unified framework. To fill this gap, this study investigated the mediating role of psychological empowerment in the relationship between school support and vocational college teachers’ digital literacy. Our findings indicate that school support not only directly and positively predicts teachers’ digital literacy but also enhances their psychological empowerment, which in turn stimulates their motivation and capacity to actively learn and apply digital technologies. On the other hand, this result is also strongly supported by Social Cognitive Career Theory (SCCT) (Lent et al., 1994) and Psychological Empowerment Theory (Spreitzer, 1995). According to SCCT, environmental support serves as a key antecedent variable for enhancing individuals’ self-efficacy and behavioral commitment. In an environment with adequate school support, teachers are more likely to obtain technical training opportunities and collaborative resources from peers, thereby strengthening their belief that “I can effectively utilize digital technology,” and subsequently stimulating their willingness and behavior to actively learn and practice digital skills. Psychological Empowerment Theory further explains this mechanism, positing that individuals who perceive organizational trust, value recognition, and decision-making autonomy are more committed to their roles and motivated to pursue excellence. In the context of vocational education, teachers facing rapidly evolving digital tools and complex industry–education integration demands may resort to passive coping or experience technology anxiety when relying solely on external training. However, when schools convey clear policy signals—such as “you are capable, you are trusted, and your innovation is valued”—teachers are more likely to internalize this support. This fosters a sense of autonomy and purpose, motivating them to proactively explore and innovate with digital technology.

In summary, beyond providing external support, vocational colleges should empower teachers internally through organizational practices. This includes giving teachers a voice and decision-making power in digital reforms, fostering a culture that encourages the active and confident use of technology, and enhancing teachers’ perceived value of digital teaching through project-based learning and collaboration, thereby effectively activating the intrinsic motivation for digital literacy among vocational college teachers.

### 5.3 The mediating role of professional identity in relationships

Research findings indicate that professional identity plays a significant mediating role between school support and the digital literacy of vocational college teachers. Specifically, when schools offer institutional guarantees, resource investment, and cultural incentives, teachers’ sense of value, belonging, and mission as “high-quality technical and skilled talent cultivators” in their professional roles is enhanced. This strengthened professional identity further stimulates their intrinsic motivation to actively adapt to the teaching changes in the digital age, prompting them to integrate and innovate digital technologies in teaching practices.

This finding aligns with previous research (Wang and Ye, 2025) and further supports social identity theory (Tajfel and Turner, 1979), which posits that individuals’ identification with their professional group profoundly influences their behavioral commitment and willingness for professional development. Within the context of vocational education, teachers frequently face external pressures such as “marginalized academic status” (Habibi et al., 2023) and “insufficient social recognition” (Fan and Liu, 2024). Without organizational support, they are prone to professional burnout or identity ambiguity. However, when schools explicitly assign weight to digital literacy in the evaluation of professional titles and include teachers in the core team of digital professional development, this support reinforces their identity as leaders in modern vocational education. Grounded in this psychological foundation, educators are more inclined to view digital technologies as tools for fulfilling their educational mission rather than as additional burdens.

### 5.4 The serial mediating effect of psychological empowerment and professional identity

The results reveal that psychological empowerment and professional identity serve as serial mediators in the relationship between school support and vocational teachers’ digital literacy. Specifically, when vocational teachers experience enhanced psychological empowerment through external school support, this empowered psychological state can further promote the teachers’ value recognition and emotional attachment to the identity of vocational educators, leading them to view mastering and applying digital technologies as an inherent requirement for fulfilling their educational mission. This progressive pathway from “external support” to “psychological empowerment” and then to “identity internalization” effectively enhances digital literacy.

This finding is consistent with the work of Ding and Xie (2021), who demonstrated that psychological empowerment and professional identity are significantly positively correlated among 650 primary and secondary school teachers in rural China. This finding can also be explained by social cognitive career theory, which posits that individuals’ belief in their professional competence (i.e., self-efficacy, a key component of psychological empowerment) is a core prerequisite for forming a professional identity. Psychological empowerment, a multidimensional construct encompassing self-efficacy, autonomy, and perceived influence, strengthens when vocational teachers feel “I can do it” and “I have a voice” in digital teaching. This, in turn, solidifies their professional identity as trainers of technical skills for the new era. Teacher identity construction theory further supports this view, positing that organizational empowerment fosters professional initiative, thereby promoting the internalization and stabilization of professional identity (Beauchamp and Thomas, 2009).

### 5.5 Multi-group comparison of teachers with different professional titles

This study employed MGSEM and the Bootstrap method to examine differences in the pathway of “school support → psychological empowerment → professional identity → digital literacy” among teachers with different professional titles in vocational colleges. The results show that the theoretical model demonstrated robust measurement invariance and structural fit across all three groups. However, the mechanism through which school support influences teachers’ digital literacy varied significantly across professional titles.

Firstly, regarding the mediating path of psychological empowerment, only senior-level teachers achieved a significant level, and its effect size (or path coefficient) was significantly stronger than that for middle-level teachers. This suggests that for senior teachers, school support enhances digital literacy primarily by boosting their psychological empowerment. Secondly, on the mediating path of professional identity, significance was attained only for intermediate-level teachers. However, all cross-group difference confidence intervals included zero, indicating that while this path was significant for intermediates, its effect size was not statistically greater than in other groups. Finally, the serial mediating path was significant only for junior-level teachers, with an effect size significantly stronger than that for intermediate-level teachers. This indicates that junior teachers rely more on the complete psychological serial of “empowerment → identity recognition” to improve their digital literacy. In summary, the developmental mechanisms of digital literacy among vocational college teachers exhibit distinct stage-specific patterns: junior-title teachers are characterized by serial mediation, intermediate-title teachers by identity-driven pathways, and senior-title teachers by psychological empowerment.

Huberman’s (1993) teacher career cycle theory helps explain the professional title heterogeneity in this study. The theory posits that teachers’ professional focuses, psychological needs, and organizational dependencies evolve across career stages—differences further amplified by the industry-education integration inherent in vocational education. Junior-title teachers, in the “survival and identity construction” stage, must adapt to teaching norms while transitioning from “academic professionals” to “industry practitioners.” They require school support that simultaneously builds confidence (psychological empowerment) and a sense of value (professional identity) to convert external resources into digital literacy. This explains the significance of the serial mediation pathway: only through this dual-track empowerment can novice teachers embrace digital transformation. Intermediate-level teachers, having overcome survival anxiety, face occupational burnout from repetitive work. Their core need is to restructure meaning by solving real-world industrial problems. School support that strengthens their professional identity, such as participation in enterprise innovation or digital course development, can effectively activate their motivation to improve digital literacy. Senior-level teachers, as professional leaders, seek to shape industry discourse and achieve self-actualization. They need not basic recognition but opportunities to lead industry standards through digital technology. When granted authority to lead industrial colleges or formulate technical norms, their sense of empowerment directly translates into enhanced digital literacy. The divergence of these three pathways thus reflects the deep interaction between role reconstruction, evolving needs, and industry integration throughout a teacher’s career lifecycle.

### 5.6 Implications and limitations

Theoretically, this study contributes to the literature in the following ways. Firstly, by integrating social cognitive theory, self-determination theory, and conservation of resources theory within the context of vocational education, this study reveals the multi-level formation mechanisms of digital literacy, thereby enriching the theory of teacher professional development. Secondly, examining two distinct mediating variables simultaneously helps uncover the “psychological transformation pathway” and “identity recognition pathway” through which school support influences digital literacy, deepening our understanding of these mediating mechanisms. Finally, focusing the research perspective on the specific group of vocational college teachers contributes to advancing digital literacy research for educators from a general to a typological approach. The practical significance is twofold. The findings provide vocational college administrators with a clear intervention pathway—by enhancing school support, both teachers’ psychological empowerment and professional identity can be strengthened simultaneously, thereby systematically improving their digital literacy. Additionally, the results further reveal differences in the mediating pathways among teachers with different professional titles, offering empirical evidence to support the development of a hierarchical and differentiated system for enhancing teacher digital literacy.

This study has several limitations. Firstly, the data originate from a cross-sectional questionnaire survey, which can reveal associative mechanisms among variables but struggles to establish causal sequencing. Future research could employ longitudinal tracking designs or experimental intervention studies to dynamically examine the evolutionary process of teachers’ digital literacy formation. Secondly, although the sample covers vocational colleges across eastern, central, and western China, demonstrating good regional representativeness, the analysis did not deeply explore region as a moderating variable. In fact, differences exist in industrial foundations, the depth of school-enterprise cooperation, and educational digital investment among different regions. Future studies could conduct regional stratification or multi-group comparisons to reveal the shaping effect of the institutional and ecological context on teacher development mechanisms. Finally, the measurement of digital literacy relies primarily on teacher self-assessment. Future studies could integrate objective indicators such as classroom observations and analyses of digital teaching artifacts to achieve triangulation through multiple data sources.

## Data Availability

The original contributions presented in the study are included in the article/Supplementary material, further inquiries can be directed to the corresponding author.

## Footnotes

1
https://www.wjx.cn
